# Oil/Salt Use Assessment of Chinese-Style Canteens Based on Consumers’ Perception of the Nutrition Environment

**DOI:** 10.3390/nu15204321

**Published:** 2023-10-10

**Authors:** Yue Han, Zhihong Fan, Tongfeng Li

**Affiliations:** College of Food Science & Nutritional Engineering, China Agricultural University, Beijing 100083, China; hanyue@cau.edu.cn (Y.H.); 2019319010419@cau.edu.cn (T.L.)

**Keywords:** nutrition environment, perception, oil and salt, Chinese-style canteens, KAP

## Abstract

Excess cooking oil and salt use in catering services contributes to obesity and cardiovascular disease, but the assessment of oil/salt use has been a challenge in nutrition environment measurement. We conducted a knowledge, attitude, and practice survey on 250 respondents in five university canteens at China Agricultural University, Beijing, China. Using on-site tools including a newly developed Likert scale and the previously tested Oil–Salt Visual Analogue Scale (OS-VAS), the respondents were asked to evaluate their personal taste, their impression of the oil/salt status of canteen dishes, and their attitude toward oil/salt reduction. Data analysis showed that gender and self-image of body shape had a significant impact on KAP scores and the impression of the oil/salt environment. The respondents’ taste preferences correlated with their perception of oil and salt, but knowledge and attitude were not directly related to scores on oil and salt, while weight status was related to oil and salt scores. The Likert scale-based assessment could work but was not as effective as the OS-VAS in distinguishing the differences among the selected canteens. These results indicate that the quality of the nutrition environment in catering services needs to be comprehensively evaluated with an objective evaluation of raters and a subjective evaluation of consumers.

## 1. Introduction

The association between unhealthy dietary patterns and chronic non-communicable diseases (NCDs) has been widely explored [1,2]. A high intake of sodium, low intake of whole grains, and low intake of vegetables are regarded as the leading dietary risk factors globally and in many countries [3]. Eating outside one’s home is regarded as a risk factor for obesity and NCDs due to the consumption of unbalanced food groups and excess consumption of fat and salt [4,5].

Food consumer behavior can be described as the process of interaction between a population and its nutrition environment. Individual dietary choice is affected by a range of environmental determinants, including the food and nutrition environment at the community, national, and global levels [6]. The nutrition environment, also known as the food environment, is a conceptual model first proposed for understanding and improving the environmental variables associated with the food behaviors of people in certain communities [1]. The nutrition environment model posits that both environmental and personal factors can have a substantial impact on diet, thereby affecting the risk of overweight, obesity, and chronic diseases. Therefore, the nutrition environment plays a crucial role in dietary behavior and energy intake [1]. People should be aware that the appointed food providers or catering services in their vicinity can have an important impact on the health of regular diners.

The environmental variables related to the nutrition environment of catering services are composed of factors that indicate the nutrition quality of the food provided, the price policy, the adjustable services, and nutrition-related information. These factors can be assessed using tools designed for the objective assessment of the nutrition environment [2], such as the Nutrition Environment Measures Survey in Restaurants (NEMS-R), the Freedman’s comprehensive dining survey [3], and the Full Restaurant Evaluation Supporting a Healthy (FRESH) Dining Environment Audit [7].

While objective assessment methods of the nutrition environment have been widely used, the perception of the population on their nutrition environment, an important factor that may affect consumer food behavior, has not been paid enough attention. In 2015, a model and plan for the Perceived Nutrition Environment Measures Survey (NEMS-P) [8] was proposed, which focused on the evaluation of diet-related behaviors and habits, personal perception and interpretation of the nutrition environment, and socioeconomic and physical differences in terms of dietary behavior. NEMS-P has been verified and applied in the cultural background of the United States [8] and European countries [9]. As an emerging perspective in nutrition environment assessment, the model of NEMS-P deserves further investigation and application in more diverse dietary cultural settings, such as in Asian countries. However, due to differences in dietary habits and culture, the NEMS-P scoring system cannot be directly applied to Asian food consumers.

Chinese cuisine includes many cooking techniques and the use of seasonings, which may have different impacts on the oil and salt intake of diners. For example, steaming and steam frying (a quick stir-fry with a small amount of oil followed up covering with the lid, and the food is heated by steam in the pot) can cook dishes with a minimum amount of oil, while deep frying can greatly increase the fat content of food. The pre-treatment of food materials with salt or soy sauce introduces excess salt to cooked dishes, while the addition of salt and soy sauce after frying can reduce the use of salt on the basis of the same level of acceptability.

In 2015, China’s daily per capita salt intake reached 10.5 g [10], more than twice the recommended intake (5 g per day) by the World Health Organization and the Dietary Guideline of China (2022). With the efforts of China’s salt reduction policies and actions, the daily per capita salt intake decreased to 9.3 g in 2020 [11]. However, excessive salt intake still ranks first among cardiovascular risk factors in the diet of Chinese residents [12], leading to overweight and obesity by increasing appetite [13], eliciting insulin resistance [14], and posing a huge threat to health. There is also research evidence that excessive salt intake can reduce immunity [15] and is associated with an increased risk of diseases, such as osteoporosis, kidney stones [16], and gastric cancer [6].

According to the latest data, the per capita daily cooking oil consumption in Chinese households reached 43.2 g, with over half of the residents consuming more than 30 g of cooking oil [11], far exceeding the recommended daily edible oil intake standard of 25–30 g in the Chinese Dietary Guidelines (2022) [17]. Although most cooking oils in China, such as soybean oil, low-erucic rapeseed oil, and peanut oil, contain a high percentage of unsaturated fatty acid, excessive energy intake from edible oil is closely related to overweight and obesity, as well as an increase in blood glucose and blood pressure [18]. Curbing the amount of cooking oil has become one of the major tasks of health promotion programs in China [19].

In China, most college students live on campus and depend heavily on the food provided by the canteens on their campus. They are not allowed to cook in their dormitory. Thus, we considered that canteens at a university could be a perfect research sample for assessing the perception of the nutrition environment, focusing on the perception of the status of oil and salt use.

However, the assessment of edible oil and salt use in catering services remains a difficult task since it is difficult to analyze all the dishes offered by catering services. Based on consumers’ personal perceptions, we developed a visual analog scale for assessing oil and salt (OS-VAS) use in canteens [20], which was easy to apply in catering settings with regular consumers and patrons.

We hypothesize that in university students, the perception of oil/salt use would be affected by nutrition knowledge, attitude, and practice (KAP), as well as weight-related motivation. In this study, we tried to investigate the respondents’ perception of oil and salt use in the canteen, their KAP on oil/salt use in canteens, and the possible relationship between their subjective assessment and their KAP scores. 

In this study, we newly developed a short Likert-scale questionnaire to investigate the eaters’ impression of oil/salt use and compared the subjective ranking in the questionnaire with the VAS tool with respect to their resolution in the oil/salt assessment.

Since an evaluation of the nutrition environment in terms of oil/salt use in a Chinese food culture background has yet to be explored, we hope this study can contribute to the nutritional quality assessment of Chinese-style catering services from the perspective of consumers’ perception.

## 2. Methods Overview

Based on the actual situation in China and the concept of NEMS-P, we conducted a survey of consumers’ knowledge, attitudes, and perceptions and compared two evaluation tools we developed for the consumers’ subjective assessment of the amount of oil/salt use. Taking the local dining scenarios and consumer dietary preferences into account, we attempted to establish a connection with the consumer assessment of the nutrition environment in the canteens. We categorized the dishes into 6 groups according to cooking methods and used a 5-level Likert scale [21] to rate the consumers’ preference for them. Five Chinese-food university canteens in the east campus of China Agricultural University, where most full-time students regularly dine, were chosen as the research setting.

### 2.1. The Inclusion and Exclusion Criteria for Participants

Healthy university students and staff were randomly selected and face-to-face interviewed at the time of lunch or dinner in any one of the selected university canteens. The inclusion criteria for respondents were those who had studied or worked at China Agricultural University for at least one year, had dining experience in all five selected canteens, had no diagnosed gastrointestinal disease or other serious diseases, ate meals regularly every day, and did not exclude certain categories of dishes in the canteens. Visitors and students who did not live on the campus were excluded from the respondents to rule out any possible confounders.

In order to avoid possible bias caused by gender and the sample size of respondents in different canteens, we randomly selected 50 respondents from each canteen and made the gender ratio close to 1:1.

### 2.2. Selection of Investigation Venue and Collection of Basic Personal Information

The selected five canteens were recorded as Canteen A, Canteen B, Canteen C, Canteen D, and Canteen E. The canteens were located inside the campus and were managed by the university. The food served included staple food, Chinese salad dishes, cooked animal-origin foods, cooked vegetables, fruits, and drinks, etc., which were similar to the food that could be obtained at home. The students and staff could choose their favorite staple food and dishes from any of the canteens. After completing payment at the window, they could eat in the canteens or pack their food away. Prior to the present investigation, we had conducted an assessment of the nutrition environment of these five canteens, fully understanding their service policies and the healthiness of the food. According to our previous survey, there were differences with respect to the nutrition environment among the canteens, and we believed that the scores could be improved by the collaborative effort on both the canteen side and the diners’ side. 

We added some questions about the respondents’ basic attitudes in the ‘Respondent Information’ section, such as their intention to gain or lose weight, their attitude toward using diet to prevent or control diseases, and their concern about the impact of food on disease prevention and control.

### 2.3. Knowledge, Attitudes, and Practices Related to Salt and Edible Oil Intake

In the section “Knowledge related to salt and edible oil intake”, we compiled 13 “should know” questions on salt and edible oil use and the health consequences of overusing them. One point was given for each correct answer to the question. No score was given for any incorrect answers.

When investigating respondents’ attitudes toward salt and edible oil intake, we obtained results based on whether they were willing to choose to reduce their intake of salt and edible oil in order to control their weight, whether they were willing to choose to reduce their intake of salt and edible oil in order to prevent diseases, and the importance they attached to taste, nutrition, safety, price, convenience, weight control, and disease prevention when dining in the canteens. For the question ‘Are you willing to reduce salt/edible oil intake in order to control weight/prevent diseases’, the answer ‘yes’ received one point, while the other answers did not receive any points. For the question of ‘How much are you concerned about nutrition/safety/weight control/diseases prevention when dining in the canteen’, the answer ‘very concerned’ was given 2 points, the answer ‘relatively concerned’ was given 1 point, and the answer ‘not mind’ was not given a score. The highest score for this section was 12 points.

When investigating the behaviors of diners, we first asked them about their preference for the saltiness and greasiness of the dishes. The degree of preference was measured using five options: “extreme preference for salt/oil”, “preference for salt/oil more”, “normal taste”, “preference for salt/oil less”, and “extreme dislike of salt/oil”. Due to the selection of the survey location in Chinese-style canteens, we hypothesized multiple scenarios based on the characteristics of Chinese food to understand the dietary behaviors of the respondents in different situations. In this section, we rated the frequency of respondents ordering takeout or dining out, as well as their actions for reducing oil and salt intake during meals. We divided the frequency of ordering takeout or dining out into four options: almost every day, 3–5 days per week, 1–2 days per week, and 1 day or less per week, corresponding to 0 points, 1 point, 2 points, and 3 points. When we evaluated the behavior of respondents who requested less oil or salt when ordering takeout or dining out, and respondents who would judge the amount of oil and salt added to a dish based on its appearance to determine their dining choices, we rated the respondents based on their frequency of the behavior, which meant they received 3 points for ‘often’, 2 points for ‘sometimes’, 1 point for ‘occasionally ‘, and 0 points for’ never ‘. Due to the possibility of excess oil and salt in sauce or soup foods, respondents who chose to have less or no sauce or soup during meals received 1 point, while respondents who chose other options did not receive any points. The behavior ‘Rinse oil with hot water or free soup’ would remove more oil than ‘Drain the oil when eating’, but both were positive behaviors to reduce excessive oil intake. Therefore, we assigned 2 and 1 points to the respondents who chose one of these two options, while those who did not care about excessive oil in the dishes would not receive a score. The highest score for this section was 14 points.

### 2.4. Perception of the Nutrition Environment of Chinese Style Canteens

**Figure 1:**
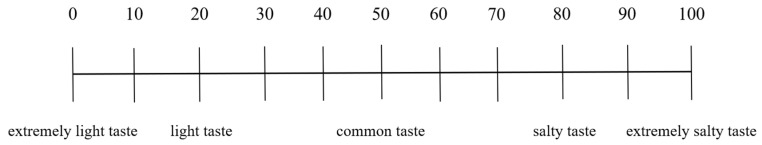
Personal dish saltiness preference score.

**Figure 2:**
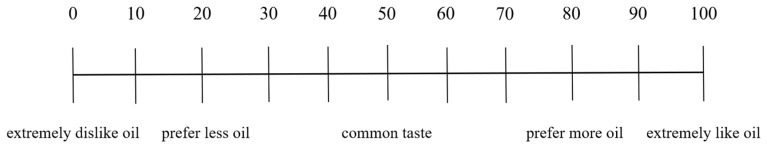
Personal oil consumption preference score.

**Figure 3:**
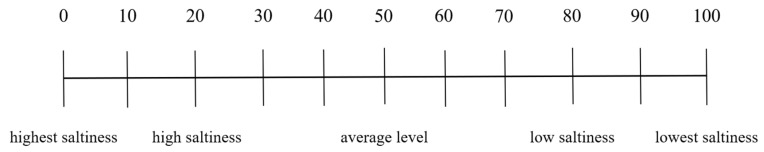
Overall salinity score of canteen dishes.

**Figure 4:**
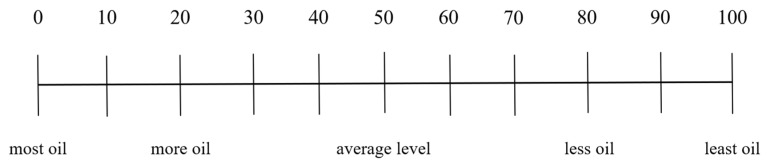
Overall oil consumption score of canteen dishes.

**Figure 5:**
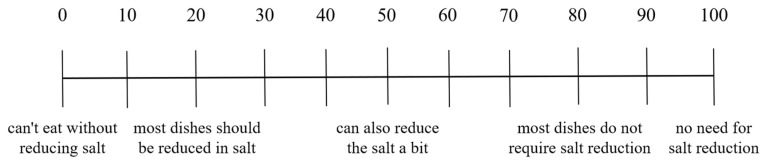
Score of salt reduction demand of canteen dishes.

**Figure 6:**
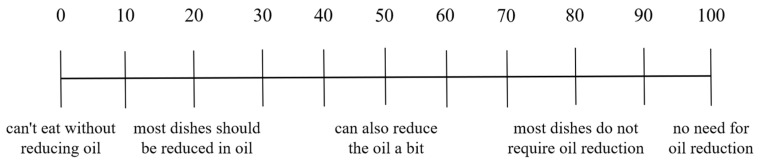
Score of oil reduction demand of canteen dishes.

In this section, we focused on the respondents’ impression of the canteens, their evaluation of the saltiness and greasiness of the canteen dishes, and their demand for reducing oil and salt. We evaluated consumers’ impression of the canteens by investigating their level of agreement with whether canteens offered light-tasting dishes and whether canteens highlighted healthy choices such as salt and oil reduction. The impression evaluation of the canteens was divided into five levels: “very disagree”, “comparatively disagree”, “unclear”, “comparatively agree”, and “very agree”. We divided the dishes in the canteens into 6 categories based on cooking techniques: cold dishes, steamed dishes, fried dishes, barbecued dishes, stir-fried dishes, and stewed dishes. Respondents used the Likert scale and OS-VAS (Figure 1, Figure 2, Figure 3, Figure 4, Figure 5 and Figure 6) to evaluate the saltiness and greasiness of canteen dishes. Similarly, when using the Likert scale to evaluate the saltiness, greasiness, salt reduction, and oil reduction needs of canteen dishes, we rated the respondents’ evaluation of the dishes as 1–5 points. The higher the saltiness/greasiness/salt reduction/oil reduction needs of the dishes, the lower the scores.

### 2.5. Data Analysis

After completing the collection of paper questionnaires, we used SPSS 22.0 to input and analyze the data.

For comparison purposes, except for genders, we also investigated the identities of the respondents and categorized them into 3 categories: undergraduate, postgraduate, and teaching and administrative staff. A frequency analysis was used to statistically analyze the respondents’ answers, the chi-square test was used to analyze respondents’ attitudes and preferences toward dining, and an analysis of variance was used to deal with scores obtained for respondents from different locations, identities, and genders who answered questions about oil- and salt-related knowledge. In addition, we used a one-way ANOVA and independent sample *t*-test to explore the impact of respondents’ identity, gender, and weight-related intention on KAP scores. We used a one-way ANOVA to explore the correlation between respondents’ impressions of their own weight and their scores using VAS and Likert scales.

For the scores given by respondents on the Likert scale or OS-VAS for salinity, greasiness, and salt reduction requirements and oil reduction requirements of canteen dishes, when analyzing the OS-VAS or Likert scale scores for different canteens, in order to avoid the impact of respondents’ personal preferences on the results, we used the ratio obtained by dividing the respondents’ scores for canteen oil or salt by their personal preference scores for oil or salt as the scores for the canteen.

## 3. Results

### 3.1. The Basic Information on the Respondents

The majority of respondents participating in this survey were undergraduate and graduate students, with teaching and administrative staff accounting for only 6.4% of the total number. We paid attention to the gender balance of the respondents during the survey process, and the numbers of males and females in the 250 respondents were roughly equal, accounting for 50.4% and 49.6%, respectively. Nearly half of the respondents were satisfied with their weight, but one-third of them had the motivation to lose weight. Two-thirds of the respondents had most of their meals in university canteens (Table 1).

### 3.2. Knowledge Related to Salt and Edible Oil Intake

The percentage of correct answers for knowledge related to salt and oil intake is shown in Table 2. Most respondents had a clear understanding of common diseases caused by excessive salt intake, such as hypertension, coronary heart disease, kidney stones, and overweight. 

The scores in this section did not show significant differences among identities, survey locations, or between genders.

### 3.3. Attitudes Related to Salt and Edible Oil Intake

Most respondents had positive attitudes toward salt/oil reduction. At the same time, most of them also paid considerable attention to taste, nutrition quality, and safety when dining in the canteens (Table 3).

We cross-analyzed their self-image of weight status and their willingness to reduce salt and edible oil intake and found a significant positive association between the self-image of weight status and salt/oil reduction-related attitudes. Compared with those who believed they were slim or had a suitable weight, the respondents who believed they were overweight had a significantly higher proportion of positive answers with respect to a willingness to reduce salt and edible oil intake to control their weight (Table 4). However, we did not observe a significant correlation between respondents’ identity and attitude toward oil/salt reduction.

### 3.4. Practices Related to Salt and Edible Oil Intake

In this section, we investigated the respondents’ taste preferences for salt and edible oil, the frequency of dining or ordering takeout outside the canteen, whether they took oil and salt reduction actions during dining, the types of dishes they liked to eat, and which canteen(s) they preferred to go to for meals (Table 5). Nearly one-fourth of the respondents described themselves as light-taste in terms of salt and over 30% of them preferred less oil. When encountering dishes with excessive oil, two-thirds of them chose to drain the excess oil before eating.

We combined the respondents’ taste preferences for oil and salt to analyze their preferences for different types of dishes. There were significant differences in the types of dishes favored by respondents with different preferences for oil and salt flavors. People with a salty taste tended to prefer fried and barbecued dishes, while those with a lighter taste tended to prefer cold dishes and steamed dishes. People with a preference for cooking oil tended to prefer fried and barbecued dishes, while those who liked low-fat dishes tended to prefer steamed and cold dishes (Table 6 and Table 7).

### 3.5. KAP Scores Related to Salt and Edible Oil Intake

We analyzed the KAP scores of respondents from the perspectives of their identity, gender, and impression of weight. There were significant differences between genders in attitude, practice, and KAP scores. Respondents with different perceptions of their weight also scored significantly differently in terms of attitude (Table 8).

### 3.6. Perception of the Nutrition Environment

We used Likert scales to survey respondents’ basic impressions of the canteens. They were asked to choose an option from five levels of identification: “very disagree” to “very agree”. When recording scores, we evaluated the canteens based on the respondents’ answers and the difficulty of obtaining healthy food from the canteens as 1–5 points (Table 9). About 44% of the respondents reported that they had difficulties finding foods that could meet the salt reduction recommendation, and 64% of them gave low scores on the canteens’ efforts to highlight healthy choices. Most respondents did not agree with the description that oil/salt-reduced food was more expensive.

Both the Likert scale and OS-VAS could distinguish the respondents’ different impressions of the canteens and their ratings of the dishes in terms of oil and salt. However, the OS-VAS achieved a more pronounced distinction among different canteens (Table 10).

We analyzed the assessment results of oil and salt in canteen food among responders of different self-reported weight statuses (Table 11). It was found that overweight responders had significantly more preference for saltiness in both the Likert scale and VAS survey. Weight statuses also had significant associations with the VAS oil-reduction demand and the Likert scale greasiness preference. However, we did not find any significant correlation between knowledge, attitude, and oil/salt assessment.

## 4. Discussion

In this study, we explored a new Likert scale method for measuring the oil/salt use in canteens, in comparison to the previous OS-VAS tool, from the perspective of consumers’ perception of the nutrition environment. Unlike the earliest proposed Perceived Nutrition Environment Measures Survey (NEMS-P) [8], our focus was not on distinguishing the views of residents of different socioeconomic statuses on the nutrition environment. Instead, we attempted to develop assessment tools based on the experience and judgment of the diners’ different taste preferences and weight statuses.

We assumed that the dietary behavior of respondents was a process of interaction among rational health-related motivation, instinctive taste preference, and the nutrition environment. Since subjective judgment on oil and salt use in canteen foods might be impacted by an individual’s own strong/light taste, we adjusted the scores with the self-reported taste preference. Therefore, the quality of the nutrition environment in catering services needs to be comprehensively evaluated from two perspectives in the future: an objective evaluation of the raters and a subjective evaluation of the consumers.

Previously, surveys were conducted on knowledge, attitudes, and behaviors related to salt among Chinese people, which have clear guiding significance for formulating better strategies for salt intake and reduction [22,23]. Since our survey involved respondents’ evaluation of the amount of oil and salt added to canteen dishes, we also referred to this survey model. After examining the scores of respondents’ oil- and salt-related knowledge by survey location, identity, and gender, we found that most respondents showed high levels of attention to nutrition, weight control, and disease prevention.

However, we did not find any significant impact of knowledge, attitude, or practice on the oil/salt scoring of the respondents. A possible reason might be that the majority of respondents were students. They knew theoretically that excess salt/oil use was hazardous to health. However, because of their age, most of them had not experienced any serious diseases. Their knowledge did not alter their daily impression of food taste in canteens. The most concerned health issue in young people is weight management, as they pay much attention to their body shape. This can explain why their self-image of body weight was associated with their scoring. Compared with those who believed they were slim or had a normal weight, those who believed they were overweight were more willing to consume less salt and edible oil.

The cooking methods of Chinese cuisine are diverse, and the seasoning and sauces of dishes can be easily adjusted according to the preferences of consumers. In a consumer-oriented society, excessive amounts of edible oil and salt are a result of consumer preference, while at the same time, young consumers’ tastes are nurtured by the food environment.

In recent years, the rapid development of China’s food delivery industry and the growth in food delivery platform users among young people have had a significant impact on their health statuses [24,25]. In the present survey, the data showed that a considerable number of respondents would ask for more sauce or seasoning with oil and salt to their dishes or staple foods, which might introduce excess oil and salt into their daily meals.

At the same time, we found that the respondents’ preference for different types of dishes was related to their own tastes. The respondents who had a taste for heavy oil and salt were more likely to choose fried and barbecued dishes, while those with a relatively light taste had a higher possibility of preferring steamed and stewed dishes.

In addition to the previously developed OS-VAS, we used the Likert scale in this survey to evaluate the oil/salt status of different canteens by scoring the responders’ impressions. The OS-VAS still showed good resolution among canteens, and the respondents’ VAS evaluation of the canteen dishes was generally consistent with our previous investigation on the nutrition environment of the same five canteens [20]. The rating results of the Likert scale showed significant differences among some of the canteens, but the difference was not as sharp as we expected. It is possible that the difference in the food served blurred the judgments of the responders. Overall, the use of the Likert scale to evaluate the measures related to reducing oil and salt in canteens achieved promising results as the canteens with higher scores in nutrition environment evaluations received higher consumer impression scores. We hope the Likert tool can be improved by modifying both the scoring questions and the assignment of weight values. 

To our knowledge, the present study was the first evaluation of the nutrition environment of Chinese-style canteens from the perspective of consumer perception of the nutrition environment survey. In order to ensure the power of the survey analysis, we balanced the gender ratio and the number of respondents in each survey site and investigated the number of times respondents chose to dine in the canteens each week. The majority of respondents dined in the canteens more than 12 times a week, which made their impressions of the canteens more reliable. In addition, we developed a Likert scale survey based on consumers’ impressions of the oil/salt status of canteen food, which represents a new non-laboratory qualitative approach to assess the general status of oil and salt added to dishes in catering services. It was also a new attempt to investigate consumers’ impressions of the canteen’s nutrition environment based on different categories of dishes.

Our study also had the following limitations. Firstly, the number and location of the survey site were limited, and the sample number of respondents must still be enlarged. Secondly, it is necessary to validate and optimize the assessment tools in more Chinese-style collective feeding units. Thirdly, compared with the OS-VAS, the Likert scale scoring resulted in less effective discrimination in terms of the oil/salt status of food in different canteens. It needs further optimization in future studies. Lastly, the possible interaction between KAP and the assessment results was not clearly identified in this research.

## 5. Conclusions

We tested a new assessment tool based on diners’ perceptions to evaluate oil and salt usage status using Likert scale scores and compared the resolution of this tool with the previous OS-VAS. There was a correlation between the taste characteristics of diners and their perception of oil and salt use. It was found that the responders’ knowledge and attitude regarding oil and salt did not significantly relate to their scoring results, while their self-reported weight status did. The scoring systems developed from the perspective of consumers’ perception of the nutrition environment have the potential to be applied in on-site assessment, but they should be further validated and improved in future studies.

## Figures and Tables

**Table 1:** Description of the basic information of the respondents.

Survey Items	Number of People	Percentage (%)
Your identity		
Undergraduate Postgraduate Teaching and administrative staff	160 74 16	64.0 29.6 6.4
Your gender		
Male Female	126 124	50.4 49.6
Your body shape and weight goal		
Slim, hoping to gain weight Overweight, hoping to lose weight Suitable, no need to gain or lose weight	47 84 119	18.8 33.6 47.6
Preventing chronic diseases using diet approaches is my concerned issue		
Yes No	152 98	60.8 39.2
I care about the impact of canteens’ food on disease prevention		
Very concerned Relatively concerned Not mind	27 129 94	10.8 51.6 37.6
Your frequency of dining in the university canteens per week		
0–2 times 3–5 times 6–8 times 9–11 times 12–14 times More than 2 times a day	3 11 34 46 71 85	1.2 4.4 13.6 18.4 28.4 34.0

**Table 2:** Description of knowledge related to salt and edible oil intake.

Survey Items	Number of People	Percentage (%)
Which one is the recommended daily salt intake for adults in the “Dietary Guidelines for Chinese Residents (2022)”?		
<3 g <5 g <6 g <8 g	17 106 111 16	6.8 42.4 44.4 6.4
Which one is the recommended range of daily cooking oil intake for adults in the “Dietary Guidelines for Chinese Residents (2022)”?		
10–20 g 20–25 g 25–30 g 30–40 g	20 116 87 27	8.0 46.4 34.8 10.8
Which item in the nutrition fact table on the food package may indicate the amount of salt added?		
Protein Fat Carbohydrate Sodium	17 24 38 233	6.8 9.6 15.2 93.2
Which item in the nutrition fact table may indicate the amount of edible oil added?		
Energy Protein Fat Carbohydrate	158 27 237 38	63.2 10.8 94.8 15.2
How many kilocalories does 1 g of edible oil contain?		
4 7 9 Not sure	12 44 55 139	4.8 17.6 22.0 55.6
Which of the following health consequences may be associated with excessive salt intake?		
Hypertension and coronary heart disease Gastric cancer Overweight Bone calcium loss Disturbance of gut microbiota Kidney stones Decreased immune function Premenstrual syndrome	238 53 92 47 32 124 51 25	95.2 21.2 36.8 18.8 12.8 49.6 20.4 10.0

**Table 3:** Description of attitudes related to salt and edible oil intake.

Survey Items	Number of People	Percentage (%)
Are you willing to consume less salt in order to control your weight?		
Yes No Not sure	149 64 37	59.6 25.6 14.8
Are you willing to consume less edible oil in order to control your weight?		
Yes No Not sure	163 52 35	65.2 20.8 14.0
Are you willing to consume less salt in order to prevent diseases?		
Yes No Not sure	201 17 32	80.4 6.8 12.8
Are you willing to consume less edible oil in order to prevent diseases?		
Yes No Not sure	196 24 30	78.4 9.6 12.0
How much are you concerned about taste when dining in the canteen?		
Very concerned Relatively concerned Not mind	141 97 12	56.4 38.8 4.8
How much are you concerned about nutrition quality when dining in the canteen?		
Very concerned Relatively concerned Not mind	102 131 17	40.8 52.4 6.8
How much are you concerned about food safety when dining in the canteen?		
Very concerned Relatively concerned Not mind	150 95 5	60.0 38.0 2.0
How much are you concerned about price when dining in the canteen?		
Very concerned Relatively concerned Not mind	56 115 79	22.4 46.0 31.6
How much are you concerned about convenience when dining in the canteen?		
Very concerned Relatively concerned Not mind	75 125 50	30.0 50.0 20.0
How much are you concerned about weight control when dining in the canteen?		
Very concerned Relatively concerned Not to mind	31 143 76	12.4 57.2 30.4
How much are you concerned about diseases prevention when dining in the canteen?		
Very concerned Relatively concerned Not mind	78 128 44	31.2 51.2 17.6

**Table 4:** Statistics on the number of people who are willing to consume less salt or edible oil in order to control their weight based on their weight impression.

Impression of Weight		Consume Less Oil			*p*-Value	Consume Less Salt			*p*-Value
		Yes	No	Not Sure		Yes	No	Not Sure	
Slim	n	20	17	10	≤0.001	21	14	12	0.017
	%	42.6	36.2	21.3		44.7	29.8	25.5	
Suitable	n	76	30	13		66	34	19	
	%	63.9	25.2	10.9		55.4	28.6	16.0	
Overweight	n	67	5	12		62	16	6	
	%	79.8	6.0	14.3		73.8	19.0	7.1	

**Table 5:** Description of practice related to salt and edible oil intake.

Survey Items	Number of People	Percentage (%)
What is your self-description of taste preference for saltiness of dishes?		
Salty taste Light taste Common taste	67 59 124	26.8 23.6 49.6
What is your self-description of taste preference for the amount of oil used in dishes?		
Prefer more oil Prefer less oil Common taste Extremely dislike edible oil Extremely like edible oil	45 77 118 4 6	18.0 30.8 47.2 1.6 2.4
How often did you dine out or order takeout in the past month?		
1 day or less per week 1–2 days per week 3–5 days per week Almost every day	119 110 16 5	47.6 44.0 6.4 2.0
Do you ask for “less oil” or “less salt” when ordering takeout or dining out?		
Never Occasionally Sometimes Often	98 111 31 10	39.2 44.4 12.4 4.0
Do you judge the amount of oil/salt added to a dish by eye when making your dining choices?		
Never Occasionally Sometimes Often	39 78 72 61	15.6 31.2 28.8 24.4
When ordering salads or Chinese-style cold dishes, what would you request?		
Add more sauce Add less sauce No request	79 92 79	31.6 36.8 31.6
When eating noodles, rice noodles, steamed vermicelli rolls, what would you request?		
Add more sauce Add less sauce No request	95 63 92	38.0 25.2 36.8
When the staff serve dishes, what would you request? Do not add the soup of the dish Add more of the soup of the dish No request	113 21 116	45.2 8.4 46.4
What will you do if the dish is soaked in too much cooking oil?		
Drain the oil when eating Rinse food with hot water to remove the oil Do nothing	163 22 65	65.2 8.8 26.0
What kind(s) of dishes do you like to eat in the canteens?		
Fried dishes Barbecue dishes Stir-fried dishes Cold dishes Steamed dishes Stewed dishes	79 117 211 55 107 123	31.6 46.8 84.4 22.0 42.8 49.2
Which canteen(s) do you like to go to for meals?		
Canteen A Canteen B Canteen C Canteen D Canteen E	144 151 125 175 85	57.6 60.4 50.0 70.0 34.0

**Table 6:** Differences in preferences for dishes among people with different saltiness preferences.

Types of Dishes		Saltiness Preferences			*p*-Value
		Light	Common	Salty	
Fried dishes	n	10	42	27	0.018
	%	12.7	53.2	34.2	
Barbecue dishes	n	23	53	41	
	%	19.7	45.3	35.0	
Stir-fried dishes	n	49	107	55	
	%	23.2	50.7	26.1	
Cold dishes	n	18	27	10	
	%	32.7	49.1	18.2	
Steamed dishes	n	37	50	20	
	%	34.6	46.7	18.7	
Stewed dishes	n	31	59	33	
	%	25.2	48.0	26.8	

**Table 7:** Differences in preferences for dishes among people with different oil consumption preferences.

Types of Dishes		Oil Consumption Preferences					*p*-Value
		Extremely Dislike	Prefer Less	Common	Prefer More	Extremely Like	
Fried dishes	n	0	19	37	18	5	0.005
	%	0	24.1	46.8	22.8	6.3	
Barbecue dishes	n	0	30	51	31	5	
	%	0	25.6	43.6	26.5	4.3	
Stir-fried dishes	n	4	61	105	37	4	
	%	1.9	28.9	49.8	17.5	1.9	
Cold dishes	n	3	21	23	7	1	
	%	5.5	38.2	41.8	12.7	1.8	
Steamed dishes	n	4	46	47	9	1	
	%	3.7	43.0	43.9	8.4	0.9	
Stewed dishes	n	1	41	50	27	4	
	%	0.8	33.3	40.7	22.0	3.3	

**Table 8:** Differences in KAP scores among respondents of different identities, genders, and impressions of weight.

Variables	Knowledge Score (Mean ± SEM)	*p* Value	Attitude Score (Mean ± SEM)	*p* Value	Practice Score (Mean ± SEM)	*p*-Value	KAP Score (Mean ± SEM)	*p*-Value
Identity								
Undergraduate Postgraduate Teaching and administrative staff	10.60 ± 0.15 10.47 ± 0.23 10.81 ± 0.45	0.784	7.69 ± 0.17 7.50 ± 0.27 8.81 ± 0.68	0.100	6.52 ± 0.19 6.82 ± 0.29 8.00 ± 0.58	0.056	24.81 ± 0.34 24.79 ± 0.60 27.62 ± 1.14	0.060
Gender								
Male Female	10.35 ± 0.17 10.81 ± 0.17	0.057	7.21 ± 0.20 8.21 ± 0.19	<0.001	6.20 ± 0.19 7.22 ± 0.23	0.001	23.76 ± 0.38 26.23 ± 0.42	<0.001
Body shape								
Slim Overweight Suitable	10.77 ± 0.25 10.51 ± 0.21 10.55 ± 0.18	0.744	6.79 ± 0.34 8.39 ± 0.25 7.59 ± 0.18	<0.001	6.98 ± 0.39 6.45 ± 0.30 6.77 ± 0.19	0.447	24.53 ± 0.71 25.36 ± 0.56 24.91 ± 0.37	0.597

**Table 9:** Description of respondents’ impression of the canteens in terms of oil/salt use.

Survey Items	Number of People	Percentage (%)
The healthfulness of the dishes in the canteen is very important to me		
Very disagree (1 point) Comparatively disagree (2 points) Unclear (3 points) Comparatively agree (4 points) Very agree (5 points)	9 15 31 118 77	3.6 6.0 12.4 47.2 30.8
There are many light tasting foods in the canteen		
Very disagree (1 point) Comparatively disagree (2 points) Unclear (3 points) Comparatively agree (4 points) Very agree (5 points)	34 85 66 51 14	13.6 34.0 26.4 20.4 5.6
It is difficult to find food that meets the salt reduction recommendation in the canteen		
Very disagree (5 points) Comparatively disagree (4 points) Unclear (3 points) Comparatively agree (2 points) Very agree (1 point)	9 57 73 84 27	3.6 22.8 29.2 33.6 10.8
Food with reduced oil and salt contents in the canteen used to be relatively more expensive		
Very disagree (5 points) Comparatively disagree (4 points) Unclear (3 points) Comparatively agree (2 points) Very agree (1 point)	19 64 100 53 14	7.6 25.6 40.0 21.2 5.6
This canteen highlights healthy choices such as oil- and salt-reduced dishes		
Very disagree (1 point) Comparatively disagree (2 points) Unclear (3 points) Comparatively agree (4 points) Very agree (5 points)	80 81 52 34 3	32.0 32.4 20.8 13.6 1.2

**Table 10:** Description of OS-VAS and Likert scale scores for the canteens.

Scoring Items	Score (Mean ± SEM)				
	Canteen A	Canteen B	Canteen C	Canteen D	Canteen E
Impression of canteens	15.56 ± 0.36 ^a^*	14.22 ± 0.46 ^b^	14.66 ± 0.38 ^ab^	15.44 ± 0.35 ^a^	13.56 ± 0.33 ^b^
VAS—Salt	1.20 ± 0.08 ^a^	0.89 ± 0.05 ^b^	0.70 ± 0.05 ^c^	1.07 ± 0.07 ^a^	0.76 ± 0.03 ^bc^
VAS—Oil	1.30 ± 0.11 ^a^	0.89 ± 0.05 ^bc^	0.71 ± 0.05 ^c^	1.00 ± 0.07 b	0.76 ± 0.04 ^c^
VAS—Salt Reduction	1.18 ± 0.07 ^a^	0.92 ± 0.05 ^b^	0.67 ± 0.05 ^c^	1.11 ± 0.07 ^a^	0.79 ± 0.04 ^bc^
VAS—Oil Reduction	1.49 ± 0.15 ^a^	0.86 ± 0.05 ^bc^	0.70 ± 0.05 ^c^	1.01 ± 0.06 ^b^	0.77 ± 0.05 ^c^
Cold dishes’ saltiness	1.06 ± 0.07	1.02 ± 0.04	0.92 ± 0.05	0.96 ± 0.06	1.03 ± 0.05
Steamed dishes’ saltiness	1.19 ± 0.05	1.29 ± 0.06	1.16 ± 0.04	1.17 ± 0.06	1.17 ± 0.05
Fried dishes’ saltiness	0.78 ± 0.05 ^ab^	0.80 ± 0.06 ^ab^	0.80 ± 0.05 ^ab^	0.88 ± 0.04 ^a^	0.72 ± 0.05 ^b^
Barbecue dishes’ saltiness	0.69 ± 0.04	0.69 ± 0.04	0.71 ± 0.04	0.73 ± 0.04	0.76 ± 0.04
Stir-fried dishes’ saltiness	0.85 ± 0.04	0.91 ± 0.05	0.85 ± 0.04	0.82 ± 0.04	0.90 ± 0.04
Stewed dishes’ saltiness	0.93 ± 0.05	0.96 ± 0.05	0.88 ± 0.05	1.00 ± 0.05	0.89 ± 0.05
Total saltiness	5.50 ± 0.22	5.68 ± 0.21	5.33 ± 0.20	5.57 ± 0.19	5.49 ± 0.18
Cold dishes’ greasiness	1.42 ± 0.07	1.34 ± 0.06	1.50 ± 0.09	1.36 ± 0.06	1.39 ± 0.08
Steamed dishes’ greasiness	1.34 ± 0.06	1.36 ± 0.07	1.42 ± 0.07	1.40 ± 0.06	1.44 ± 0.07
Fried dishes’ greasiness	0.62 ± 0.04	0.54 ± 0.04	0.62 ± 0.03	0.65 ± 0.05	0.59 ± 0.04
Barbecue dishes’ greasiness	0.72 ± 0.04 ^b^	0.72 ± 0.04 ^b^	0.86 ± 0.06 ^a^	0.77 ± 0.04 ^ab^	0.80 ± 0.05 ^ab^
Stir-fried dishes’ greasiness	0.84 ± 0.04	0.88 ± 0.05	0.98 ± 0.06	0.94 ± 0.05	0.94 ± 0.07
Stewed dishes’ greasiness	1.10 ± 0.05 ^a^	1.05 ± 0.07 ^ab^	1.10 ± 0.08 ^a^	1.11 ± 0.05 ^a^	0.92 ± 0.06 ^b^
Total greasiness	6.04 ± 0.19	5.91 ± 0.25	6.48 ± 0.32	6.25 ± 0.22	6.08 ± 0.30
Cold dishes’ salt reduction	1.20 ± 0.05	1.20 ± 0.04	1.10 ± 0.06	1.17 ± 0.07	1.19 ± 0.05
Steamed dishes’ salt reduction	1.21 ± 0.05	1.30 ± 0.05	1.32 ± 0.05	1.35 ± 0.06	1.36 ± 0.06
Fried dishes’ salt reduction	0.95 ± 0.05 ^ab^	0.96 ± 0.06 ^ab^	0.97 ± 0.06 ^ab^	1.04 ± 0.04 ^a^	0.83 ± 0.06 ^b^
Barbecue dishes’ salt reduction	0.86 ± 0.04	0.90 ± 0.05	0.86 ± 0.04	0.98 ± 0.05	0.88 ± 0.05
Stir-fried dishes’ salt reduction	0.97 ± 0.04 ^ab^	0.98 ± 0.05 ^ab^	0.99 ± 0.04 ^ab^	1.11 ± 0.06 ^a^	0.96 ± 0.05 ^b^
Stewed dishes’ salt reduction	1.05 ± 0.05 ^ab^	1.07 ± 0.06 ^ab^	0.93 ± 0.04 ^b^	1.16 ± 0.06 ^a^	0.96 ± 0.05 ^b^
Total salt reduction demand	6.24 ± 0.25	6.41 ± 0.24	6.16 ± 0.21	6.80 ± 0.29	6.18 ± 0.24
Cold dishes’ oil reduction	1.44 ± 0.06 ^ab^	1.40 ± 0.06 ^b^	1.65 ± 0.09 ^a^	1.39 ± 0.06 ^b^	1.49 ± 0.10 ^ab^
Steamed dishes’ oil reduction	1.45 ± 0.06 ^b^	1.43 ± 0.07 ^b^	1.67 ± 0.07 ^a^	1.47 ± 0.06 ^ab^	1.52 ± 0.09 ^ab^
Fried dishes’ oil reduction	0.81 ± 0.05	0.76 ± 0.05	0.83 ± 0.07	0.89 ± 0.05	0.75 ± 0.06
Barbecue dishes’ oil reduction	0.94 ± 0.04	0.89 ± 0.04	1.05 ± 0.07	0.97 ± 0.05	0.88 ± 0.06
Stir=fried dishes’ oil reduction	0.99 ± 0.04	0.97 ± 0.05	1.09 ± 0.07	1.07 ± 0.05	1.00 ± 0.07
Stewed dishes’ oil reduction	1.15 ± 0.06 ^ab^	1.11 ± 0.07 ^ab^	1.13 ± 0.07 ^ab^	1.20 ± 0.06 ^a^	0.98 ± 0.06 ^b^
Total oil reduction demand	6.77 ± 0.24	6.55 ± 0.27	7.42 ± 0.35	6.99 ± 0.26	6.62 ± 0.37

* When the lowercase letters assigned to the means in the same row are different, it is considered that there is a significant difference between the means (*p* < 0.05).

**Table 11:** Evaluation of oil and salt use by perceived weight status.

Evaluation Related to Oil and Salt (Mean ± SEM)	Impression of Weight			*p*-Value
	Slim	Suitable	Overweight	
VAS—Saltiness preference	45.72 ± 2.30	51.18 ± 1.35	53.33 ± 2.05	0.039
VAS—Greasiness preference	43.94 ± 2.28	47.17 ± 1.72	50.40 ± 2.13	0.149
VAS—Saltiness of dishes	37.49 ± 1.83	44.74 ± 1.34	43.23 ± 1.69	0.016
VAS—Greasiness of dishes	36.83 ± 2.07	40.48 ± 1.41	39.70 ± 1.73	0.383
VAS—Salt reduction demand	39.51 ± 2.16	45.47 ± 1.44	43.02 ± 1.66	0.076
VAS—Oil reduction demand	34.83 ± 2.27	42.41 ± 1.66	40.76 ± 1.62	0.031
Likert scale—Saltiness preference	2.87 ± 0.10	2.97 ± 0.06	3.20 ± 0.08	0.018
Likert scale—Greasiness preference	2.60 ± 0.09	2.87 ± 0.07	3.08 ± 0.09	0.003
Likert scale—Saltiness of dishes	15.83 ± 0.32	15.86 ± 0.18	16.02 ± 0.27	0.832
Likert scale—Greasiness of dishes	16.83 ± 0.32	16.60 ± 0.20	16.44 ± 0.29	0.667
Likert scale—Salt reduction demand	17.96 ± 0.48	18.45 ± 0.29	18.38 ± 0.33	0.659
Likert scale—Oil reduction demand	18.19 ± 0.37	18.79 ± 0.30	18.15 ± 0.26	0.229

## Data Availability

The data presented in this study are available from the corresponding author upon request.
